# Computational Study of Ferrocene-Based Molecular Frameworks with 2,5-Diethynylpyridine as a Chemical Bridge

**DOI:** 10.3390/ma3042668

**Published:** 2010-04-13

**Authors:** Feizhi Ding, Shaowei Chen, Haobin Wang

**Affiliations:** 1 Department of Chemistry and Biochemistry, New Mexico State University, Las Cruces, New Mexico 88003, USA; E-Mail: fzding@nmsu.edu (F.D.); 2 Department of Chemistry and Biochemistry, University of California, 1156 High Street, Santa Cruz, California 95064, USA

**Keywords:** electron transfer, mixed-valence system, nanoelectronics, constrained density functional theory

## Abstract

A computational study was carried out to examine the electronic and optical properties of the experimentally proposed ferrocene-based molecular diode that used 2,5-diethynylpyridine as a bridging unit. Density functional theory, time-dependent density functional theory, and constrained density functional theory were applied to investigate various aspects of the underlying electron transfer mechanism. The results not only advance our understanding of the experimental observations, but also demonstrate the usefulness of computational approaches for the design of new electronic materials.

## 1. Introduction

There has been considerable interest in the experimental and theoretical study of future nanoscale electronic devices [1,2,3,4]. Advances in technologies for the characterization and manipulation of individual molecules [5,6,7,8] and first-principles electronic structure theories for the description of electron tunneling through atomic chains or single molecules [9,10,11,12,13,14,15,16,17,18,19] have stimulated many new investigations in this area. Among these, the study of electron transport in nanoscale systems has received much attention for the purpose of understanding and designing molecular diodes, transistors, and new types of solar cells.

Complementary to experimental work, computational electronic structure studies of these functionalized nanosystems are of special importance since conventional experimental research generally involves considerable effort and the resulting microscopic mechanisms are often unclear. In order to fabricate functional devices on the basis of individual molecules, it is essential to have a thorough understanding of the electron transport process at the molecular level. Such an understanding may eventually provide fundamental insights into the design of new nanoscale electronic devices.

**Scheme 1:**
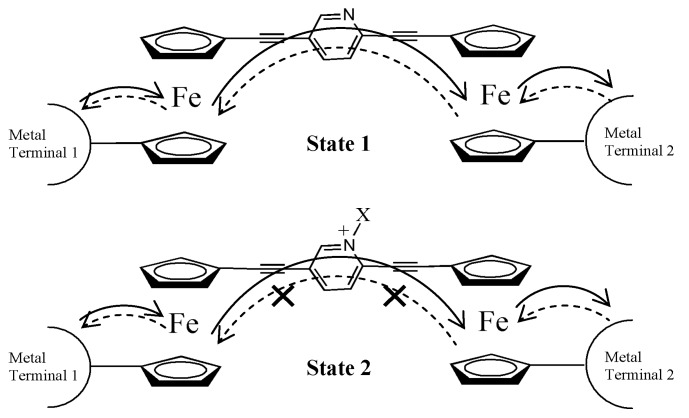
The proposed design of a ferrocene-based diode.

To date, delocalized molecular orbital networks in conjugated molecules have been proposed as an ideal design for molecular wires. A number of experimental and theoretical groups have investigated various conjugated molecules [20,21,22,23,24,25,26] and have proposed that electron transport may be readily controlled by chemical functionalization of the conjugated framework [27,28,29,30,31], for instance, by the incorporation of a metallocene-based organometallic unit [32,33,34,35]. Indeed, with the inclusion of one ferrocene moiety, it has been shown that the rate of electron transfer through the resulting framework is much higher than that through an organic conjugated system [33]. The major advantage of a ferrocene-based approach to molecular circuits is that it exhibits a lower turn-on voltage and much higher operating stability than typical organic molecules since the ferrocene/ferrocenium redox potential is considerably lower than those of organic molecules, and the redox potential can be readily tuned over a wide range by the judicious choice of substituents on the rings of the ferrocene moiety. In fact, a great deal of work has been done in the investigation of the impacts of varied structural parameters on the rate of electron transfer within materials possessing multiple ferrocene sites. For instance, Sita and co-workers [32] have proposed a new strategy for the construction of a ferrocene-based molecular diode that operates by an electron-hopping mechanism. Key to this design was the implementation of the asymmetric 2,5-diethynylpyridine bridging unit that serves to reversibly switch the system between two states (Scheme 1). It was predicted that by adding a positive charge to the nitrogen atom of the pyridyl ring, the two ferrocenyl groups would possess distinctly different redox potentials and thus create a barrier for forward electron transfer. To rationalize this design, a series of model compounds were synthesized and characterized by spectroscopic and electrochemical methods [32].

**Figure 1:**
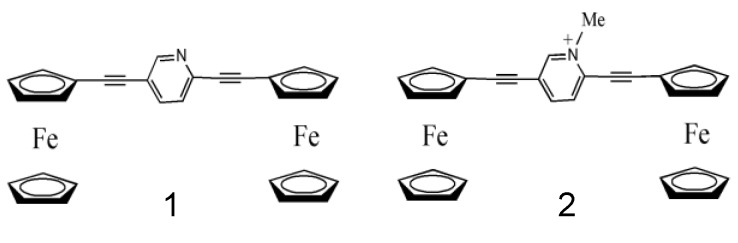
Structures of model compounds 1 and 2.

To facilitate a better understanding of the fundamental mechanism of electron transfer through this type of ferrocene-based nanoelectronics, we carried out a systematic computational study of the electronic structures, optical transitions, and electronic couplings between two ferrocenyl groups for model compounds 1 and 2 that are depicted in Figure 1. The primary purpose of our work is to elucidate the relationship between the property of the 2,5-diethynylpyridine bridging unit and the effective electronic communication of the overall system, which follows closely the previous experimental work of Sita and co-workers [32]. It should be noted, however, that this is only a necessary condition for the efficient electron transport through the metal-molecule-metal heterojunctions. A more complete modeling of such transport processes will need to include the alignment of the molecular frontier orbitals with the Fermi levels of the metals, the specific linkage of the molecule to the metal leads, and the effect of molecular vibrational motions. Such a study will be pursued in future work. In the following sections we first describe the electronic structure methods employed in our computational study. Then we present detailed analyses of our results, *i.e.,* geometries, molecular orbitals, electronic transitions that are responsible for the optical characteristics, and the electronic coupling/communication between donor and acceptor states. In the conclusion, we discuss the implication of our computational study in future experimental studies.

## 2. Computational Methods

Three computational methods were applied in this work to study the electronic and optical properties of model compounds 1 and 2, as well as other relevant compounds. Standard density functional theory (DFT) was used to study the equilibrium properties of these compounds and the characteristics of their frontier orbitals. The time-dependent density functional theory (TDDFT) was employed to examine the photoinduced electronic transitions in the model compounds and to compute the optical spectra in the ultraviolet-visible (UV-vis) range. These two well-documented computational approaches will provide structural and spectroscopic data that are directly comparable to experimental results. Furthermore, they may provide detailed electronic structural information, which can be used to elucidate the corresponding microscopic reaction mechanism.

To investigate bridge-mediated electron transport in the compounds, constrained density functional theory (CDFT) [36] was employed to define the charge-localized donor/acceptor diabatic states and to calculate the electronic coupling matrix element (or transfer integral) for the underlying electron transfer reaction. The basic idea of the CDFT approach is to impose an external constraint in the Kohn-Sham-like variation via the method of Lagrange multiplier, *i.e.*, adding an effective potential *V_c_w_c_*(r) to the Hamiltonian [36]. The resulting ground-state density satisfies specific density constraints, *i.e.*, ʃ*w_c_*(r)ρ_c_(r)dr = *N*_c_, where *w_c_*(r) is the operator that defines the property of interest. To study the electron transfer processes in the model compounds in this paper, this constraint can be defined as the charge difference (Δq) between the two ferrocenyl groups: Δq = −1 for the donor state and Δq = +1 for the acceptor state. Similar to the standard DFT method, a self-consistent procedure is used to find the minimum energy, the electronic density, and the constrained potential (the Lagrange multiplier *V_c_*) within the CDFT framework [36]. This defines the two (approximate) diabatic states for the donor and the acceptor in the electron transfer model and the resulting two-state Hamiltonian matrix. After appropriate orthogonalization (e.g., Löwdin orthogonalization) of the two diabatic states, the electronic coupling matrix element can be readily evaluated.

The DFT and TDDFT calculations were carried out by using the quantum chemistry program package Gaussian 03 [37], whereas the CDFT calculations were performed with a modified version of the quantum chemistry program NWCHEM [38]. In all the simulations, the B3LYP hybrid functional, which includes the Becke three-parameter exchange [39] and the Lee, Yang, and Parr correlation functionals [40], were employed. The LanL2DZ basis sets [41] were used for the element Fe, whereas the 6-31G** series of basis sets [42] were used for other elements such as C, N, Si and H. By employing these mixed 6-31G**/LanL2DZ basis sets, the geometry of each model compound was fully optimized at the ground electronic state in the DFT calculation. For calibration purpose, larger basis sets, 6-311++G** for C, N, Si, H and 6-31G* for Fe, were also employed in the DFT geometry optimization of the model compounds. The resulting structures and relative energies were similar to those obtained by using the smaller 6-31G**/LanL2DZ basis sets. Thus the latter, smaller basis sets were used in the calculations reported in this paper. The UV-vis absorption maxima were calculated using the TDDFT method in both the CH_3_CN and CH_2_Cl_2_ solutions, where the solvents were taken into account approximately by the standard polarizable continuum model (PCM) implemented in the Gaussian 03 program package. The gas-phase optimized geometries were used in all these solution-phase calculations. For the CDFT calculation of the electronic coupling terms the solvent effects were also modeled approximately by the COSMO approach [43] implemented in the NWCHEM program package.

## 3. Results and Discussion

### 3.1. Geometries and Molecular Orbitals.

Since compounds 1 and 2 are the major components in the design of the ferrocene-based reversible switching diode, a careful analysis of their structures may provide useful insights into the mechanism of electron transport through these two bridges.

**Figure 2:**
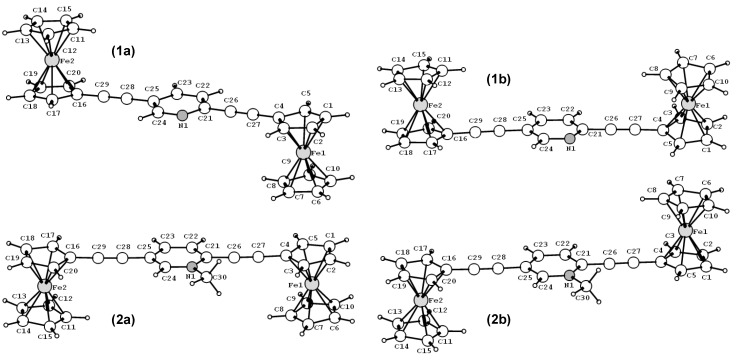
DFT optimized geometries of compounds 1 and 2.

We first optimized the two possible conformations (*cis* and *trans*) for compounds 1 and 2. Figure 2 shows the gas-phase optimized geometries for these isomers, *i.e.*, compounds 1a and 2b with a *trans*conformation, and compounds 1b and 2a with a *cis* conformation. Our calculations show that for compounds 1 and 2 the *trans*and *cis* isomers exhibit almost the same gas-phase electronic energy. In contrast, X-ray crystallographic measurements have shown that a *trans* conformation is preferred for compound 1 (Figure 1a) and a *cis* conformation for compound 2 (Figure 2a) [32], which might arise from the differences in crystal packing forces. Table 1 lists selected bond lengths and torsion angles for compounds 1a, 1b, 2a and 2b. The X-ray crystallographic data [32] are also listed for comparison. For the two isomers of each of compounds 1 and 2, the calculated bond lengths are almost the same, despite the differences in the relative orientation of the two ferrocene groups. For compound 1a that exhibits the same conformation as that determined in X-ray crystallographic measurements, the calculated bond lengths and torsion angles (the dihedral angle between the central pyridyl ring and the cyclopentadienyl rings of the ferrocenes) are in good agreement with experimental data. For compound 2a, whereas the calculated bond length is consistent with experimental data, there are some differences in the torsion angles. This discrepancy may be ascribed to the following reasons. First, in solution-phase the cationic compound 2a is accompanied by an anion PF_6_ˉ. The presence of the counter ion PF_6_ˉ may have some electrostatic effects on the crystal structure of compound 2, which can not be described by a simple gas-phase calculation that includes PF_6_ˉ (in fact, such a calculation results in a quite unreasonable structure for compound 2). The second and related point is that the theoretical geometry was obtained from the gas-phase calculation in which the crystal packing forces were eliminated. Nevertheless, the overall agreement between the theory and the experiment is quite reasonable.

From both the theoretical and experimental data it is interesting to note that there is very little difference in the corresponding carbon-carbon bond lengths between compounds 1a and 2a, despite the variation in the relative orientation of the two ferrocenyl groups. However, the torsion angle between the central pyridyl ring and the cyclopentadienyl rings of the ferrocenes is substantially different between compounds 1a with 2a. That is, for compound 1a, the pyridyl ring is nearly coplanar with the cyclopentadienyl rings of the ferrocenes, whereas for compound 2a, the central ring of the pyridinium cation bridge is tilted by ~10° with respect to the cyclopentadienyl rings.

**Table 1:** Selected theoretical bond lengths (Å) and torsion angles (Deg) for compounds 1 and 2. The experimental X-ray crystallographic data are listed in parentheses.

Bond Lengths (Ǻ)			
Compound 1a (Gas-phase Electronic Energy: ‒1419.261490)			
Fe1‒Cnt1	1.679 (1.658)	C16‒C29	1.417 (1.432)
Fe1‒Cnt2	1.674 (1.650)	C28‒C29	1.217 (1.186)
C25‒C28	1.419 (1.444)	N1‒C24	1.327 (1.356)
C24‒C25	1.414 (1.385)	N1‒C21	1.354 (1.375)
Compound 1b (Gas-phase Electronic Energy: ‒1419.261491)			
Fe1‒Cnt1	1.679	C16‒C29	1.417
Fe1‒Cnt2	1.673	C28‒C29	1.217
C25‒C28	1.420	N1‒C24	1.328
C24‒C25	1.414	N1‒C21	1.354
Compound 2a (Gas-phase Electronic Energy: ‒1458.978122)			
Fe1‒Cnt1	1.699 (1.653)	Fe2‒Cnt3	1.678 (1.638)
Fe1‒Cnt2	1.669 (1.641)	Fe2‒Cnt4	1.689 (1.655)
C16‒C29	1.408 (1.441)	N1‒C21	1.378 (1.390)
C28‒C29	1.220 (1.159)	C22‒C23	1.376 (1.376)
C25‒C28	1.408 (1.418)	C21‒C22	1.413 (1.373)
C24‒C25	1.396 (1.379)	C21‒C26	1.399 (1.457)
C23‒C25	1.423 (1.394)	C26‒C27	1.224 (1.180)
C24‒N1	1.354 (1.345)	C4‒C27	1.403 (1.425)
N1‒C30	1.477 (1.437)		
Compound 2b (Gas-phase Electronic Energy: ‒1458.978163)			
Fe1‒Cnt1	1.698	Fe2‒Cnt3	1.678
Fe1‒Cnt2	1.669	Fe2‒Cnt4	1.689
C16‒C29	1.408	N1‒C21	1.378
C28‒C29	1.220	C22‒C23	1.376
C25‒C28	1.408	C21‒C22	1.413
C24‒C25	1.396	C21‒C26	1.399
C23‒C25	1.423	C26‒C27	1.224
C24‒N1	1.355	C4‒C27	1.403
N1‒C30	1.477		
Torsion Angles			
Compound 1a			
C17‒C16‒C25‒C23	‒175.5 (‒176.2)	C3‒C4‒C21‒C22	4.7 (4.0)
C20‒C16‒C25‒C23	5.9 (2.7)	C20‒C16‒C21‒C23	‒173.8 (‒177.1)
Compound 2a			
C20‒C16‒C25‒C24	‒2.6 (10.0)	C20‒C16‒C25‒C23	178.9 (‒171.4)
C17‒C16‒C25‒C24	175.1 (‒171.1)	C17‒C16‒C25‒C23	‒3.4 (7.5)
C22‒C21‒C4‒C5	3.1 (‒11.5)	N1‒C21‒C4‒C5	‒175.4 (165.2)
C22‒C21‒C4‒C3	‒178.4 (174.4)	N1‒C21‒C4‒C3	3.1 (‒8.8)

**Figure 3:**
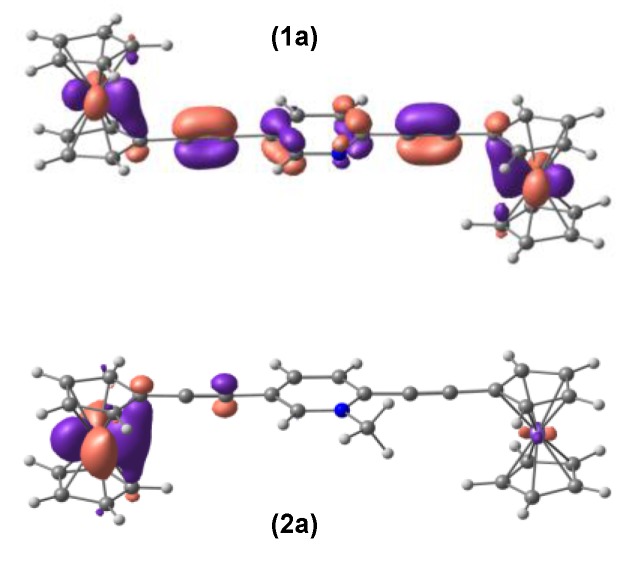
The highest occupied molecular orbitals (Contour value = 0.04) for compounds 1a (top) and 2a (bottom).

While from the X-ray crystallography data [32] alone it is difficult to ascertain whether this structural difference is caused by the electronic factors or by the different crystal packing forces, our DFT calculations suggest that it is most likely due to the presence of the positive charge on the nitrogen atom of the pyridyl ring. This is corroborated by the frontier orbital analysis. Figure 3 shows the DFT calculated highest occupied molecular orbitals (HOMOs) for compounds 1a and 2a, which display significantly different degrees of delocalization across the entire complexes. The extent of delocalization depends on the level of conjugation of the bridge group. For compound 1a, the delocalization is across the entire molecule; whereas for compound 2a, the HOMO has little contribution from the bridge and the electron density is not equally distributed in the two ferrocenyl groups. This indicates that when a positive charge is present on the nitrogen atom of the pyridyl ring the two ferrocenyl groups are not energetically degenerate, which may create distinctly different redox potentials in the two ferrocenyl groups. As pointed in the earlier experimental work [32] and our CDFT calculation in the later section, this property is crucial for the design of the molecular diode.

### 3.2. Electronic Transitions and Optical Spectra

While the geometrical analysis suggests that the presence of a positive charge on the pyridyl ring of compound 2 may differentially perturb the electronic structure of one ferrocenyl group over the other, it is still unclear whether the electron can transfer from one ferrocene to the other through the bridge, and if so, along which direction the electron transfer is favored. To address this issue, Sita *et al.* [32] carried out optical absorption spectroscopic measurements of the model compounds. The most important feature that they observed is a newly formed strong low-energy absorption band at *λ*_max_ = 540 nm (in CH_3_CN), which was ascribed to a metal-ligand charge transfer (MLCT) transition between one ferrocenyl group and the pyridyl ring in compound 2.

**Figure 4:**
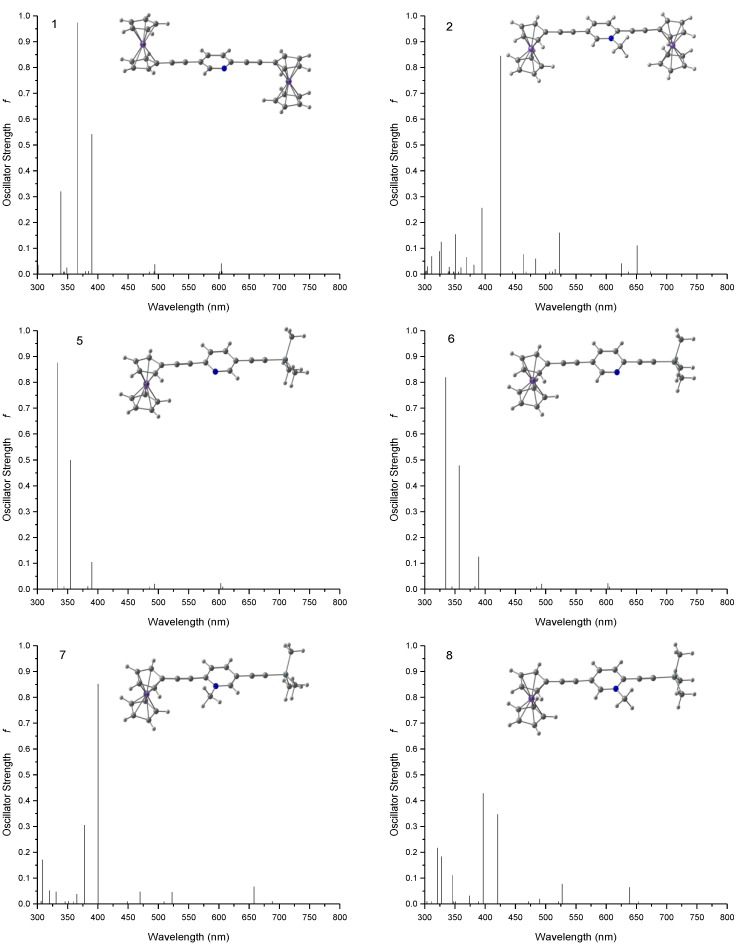
Calculated absorption spectra for compounds 1a, 2a, and 5–8 in CH_3_CN solution.

**Figure 5:**
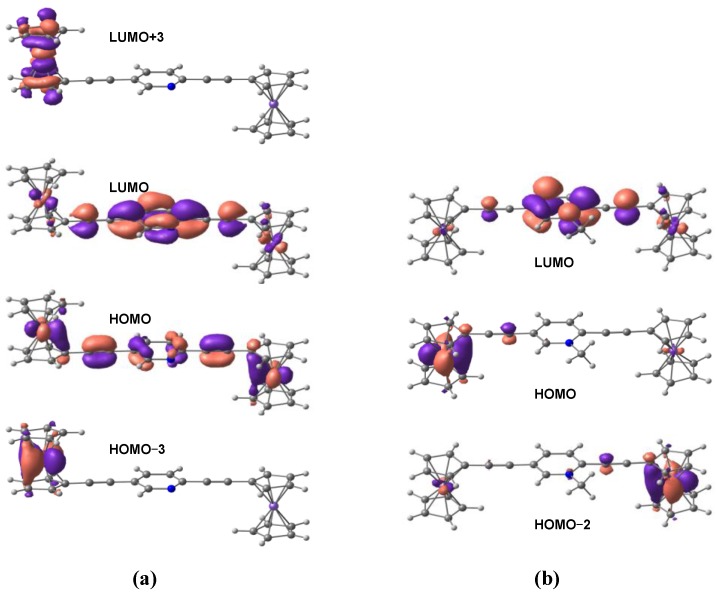
Molecular orbitals (Contour value = 0.04) involved in the electronic transitions of: (a) *λ*_max_ = 494 nm for compound 1, and (b) *λ*_max_ = 523 nm for compound 2 in CH_3_CN.

To have a better understanding of the electronic structure of the model compounds and the mechanism of electron transfer reactions through those bridging units, we have performed TDDFT calculations to study the photoinduced electronic transitions in the model compounds and to compute the UV-vis spectra in both CH_3_CN and CH_2_Cl_2_ solutions. Figure 4 shows the calculated line spectra for model compounds 1 and 2. In the realistic solution environment these line spectra will be broadened due to the solute-solvent interactions. For comparison Figure 4 also shows the spectra of four other model compounds 5−8. Compounds 5 and 6 are the two isomeric 2,5-diethynylpyridyl-derivatized monoferrocenyl compounds with one ferrocene of compound 1 replaced by the trimethylsilyl group (‒Si(CH_3_)_3_). Compounds 7 and 8 are the two isomeric 2,5-diethynylpyridinium-derivatized monoferrocenyl compounds with one ferrocene of compound 2 replaced by the trimethylsilyl group (‒Si(CH_3_)_3_). A more detailed comparison of the calculated and experimental [32] low-energy absorption maxima for the compounds under study is listed in Table 2. It can be seen that the TDDFT calculations are in semi-quantitative agreement with the experimental results. The theoretical prediction of the electronic transitions in compound 2 also shows a strong low-energy absorption at *λ*_max,calc._ = 523 nm in CH_3_CN, consistent with the experimental observation (*λ*_max,expt._ = 540 nm in CH_3_CN) [32]. The calculations also show that this transition is dominated by a HOMO → LUMO transition. As shown in Figure 5b, this HOMO → LUMO transition can be characterized as a metal-to-ligand charge-transfer (MLCT) from one ferrocene to the central pyridyl ring of compound 2. It is also interesting to note from Figure 5b that the ferrocenyl group from which the MLCT starts is further away from the charged nitrogen of the pyridyl ring. This might have some implications to the directional preference for electron transport when the 2,5-diethynylpyridine bridge is incorporated into the proposed ferrocene-based molecular diode. As can be seen from Figure 5b, the HOMO of compound 2 has little contribution from the central pyridyl ring and the electron density is localized on the ferrocene that is further away from the positively charged nitrogen of the pyridyl ring. While HOMO-2 is the nearest occupied molecular orbital that has predominant contributions from the ferrocene facing the charged nitrogen, this MO lies almost 10 kcal/mol below the HOMO, indicating that the MLCT is less likely to start from the ferrocene facing the charged nitrogen.

On the other hand, for compound 1 no strong absorption is observed in the range of 500 to 550 nm and the closest absorption maxima is 494 nm. This is a mixed transition, with a significant contribution from the HOMO‒3 → LUMO+3 transition that is characterized by a d‒d transition within one ferrocenyl group as well as a small contribution from the HOMO → LUMO transition that is characterized by a MLCT transition (see Figure 5). The considerable differences in the transition energy and strength between compounds 1 and 2 may be due to the following reasons. First, the HOMO-LUMO gap of compound 2 (2.73 eV) is much smaller than that of compound 1 (3.56 eV), making the absorption maxima red-shift from 494 nm to 523 nm. Second, the positively charged pyridyl ring of compound 2 is a better electron acceptor than the neutral pyridyl ring of compound 1, rendering MLCT energetically favorable in compound 2. Moreover, as already mentioned in the previous sub-section, the HOMO of compound 1 is delocalized over the entire molecule and the electron density is equally distributed in the two ferrocenyl groups. This indicates that the asymmetric nature of the 2,5-diethynylpyridine bridge does not lead to any significant perturbation to the electronic energies (redox potentials) of the two ferrocenyl groups.

Therefore, the above DFT and TDDFT results suggest that while the neutral 2,5-diethynylpyridine bridging unit (as appears in compound 1) may facilitate the electron conduction in both forward and reverse directions, the positively charged pyridyl ring (as in compound 2) favors the electron transfer in one direction over the other due to the energy bias between the two ferrocenyl groups. More quantitative discussion is provided below by analyzing the CDFT simulation results.

**Table 2:** Calculated lowest-energy absorption maxima for compounds 1a, 2a and 5–8. The theoretical oscillator strength (*f*) and the experimental extinction coefficient (*ε*) are also listed. The relative values of *f* and *ε* are also listed in the parenthesis.

CH_3_CN solution				
Compound	Theory		Experiment	
	*λ*_max_ (nm)	*f*	*λ*_max_ (nm)	*ε*
1a	494	0.032 (3.2)	454	3696 (2.0)
2a	523	0.150 (15)	540	10233 (5.4)
5	494	0.010 (1.0)	452	1892 (1.0)
6	493	0.010 (1.0)	446	2026 (1.0)
7	522	0.036 (3.6)	546	5810 (3.1)
8	527	0.068 (6.8)	518	4650 (2.5)
**CH_2_Cl_2_ solution**				
**Compound**	**Theory**		**Experiment**	
	***λ*_max_ (nm)**	***f***	***λ*_max_ (nm)**	***ε***
1a	494	0.029 (2.9)	456	5696 (3.0)
2a	528	0.159 (16)	582	18376 (9.7)
5	493	0.010 (1.0)	452	1993 (1.1)
6	493	0.010 (1.0)	448	1889 (1.0)
7	524	0.038 (3.8)	586	9135 (4.8)
8	535	0.069 (6.9)	564	9656 (5.1)

### 3.3. Electronic Couplings

One of the major drawbacks of the conventional DFT method is its tendency to exaggerate the delocalization of the frontier orbitals due to the self-interaction error. To have a more quantitative description of the electron transfer through the 2,5-diethynylpyridine bridging unit we have applied the CDFT approach [38] to evaluate the electronic couplings (*H*_ab_) for the model compounds 1 and 2. Table 3 lists the calculated *H*_ab_ values (in kcal/mol) in both gas phase and in a CH_2_Cl_2_ solution (the latter was modeled by the COSMO approach). The afore-mentioned DFT optimized geometries were used in all the CDFT calculations. For comparison purposes, the *H*_ab_ values for Fc−(C≡C)_n_−Fc systems are also listed.

**Table 3:** Calculated electronic couplings for model compounds.

Compound	*H*_ab_ (kcal/mol)	
	Gas Phase	CH_2_Cl_2_ solution
1	1.37	0.56
2	1.36	0.57
Fc−C≡C−Fc	2.82	1.69
Fc−(C≡C)_3_−Fc	2.00	0.97
Fc−(C≡C)_6_−Fc	1.39	0.51

As can be seen from Table 3, the calculated electronic couplings for compounds 1 and 2 are quite close to each other, but are smaller than that for Fc−C≡C−Fc, a compound that has a significant experimental splitting of the voltammetric peaks (∆*E*_½_ = 190 mV) and has been identified as a typical class II mixed-valence compound in the Robin-Day classification [44]. Based on both experimental results and our theoretical calculations, the strength of electron communication between the ferrocenyl groups in Fc−(C≡C)_n_−Fc systems was found to decrease sharply with increasing (C≡C)_n_ chain length. For instance, at n = 3, two closely spaced redox peaks (∆*E*_½_ = 60 mV) were observed in cyclic voltammetric measurement [45], indicating a borderline behavior between class I and class II characteristics. At n = 6, only a single oxidation process was observed [46], signifying a further diminishment of the electronic coupling.

Compounds 1 and 2 were also found to behave along the borderline between class I and class II compounds, as the *H*_ab_’s were estimated to be ~0.6 kcal/mol, very comparable to those of Fc−(C≡C)_3_−Fc (0.97 kcal/mol) and Fc−(C≡C)_6_−Fc (0.51 kcal/mol) in CH_2_Cl_2_. The relatively weak electronic communication may thus be difficult to resolve experimentally. For instance, in voltammetric measurements one may observe one single pair of broad voltammetric waves, instead of two well-defined ones as manifested with biferrocene. Indeed, electrochemical study of compound 1 in a 0.1 M solution of [*n*-Bu_4_N][B(C_6_F_5_)_4_] in CH_2_Cl_2_ only exhibited a single pair of voltammetric peaks.

However, for compound 2, two well-defined redox peaks were observed in voltammetric measurements (∆*E*_½_ = 161 mV), although the calculated electronic coupling for compound 2 is similar to that of compound 1. For class I/II mixed-valence compounds (*i.e.*, weaker electronic coupling) this may happen if the two ferrocenyl groups in compound 2 have distinctly different energies/redox potentials. Thus we compared the energy difference (∆E_diabatic_) between the two diabatic states (the donor and the acceptor). Since those two diabatic states were constructed by constraining the charge difference (Δq) between the two ferrocene groups: Δq = −1 for the donor state and Δq = +1 for the acceptor state, calculation of the energetic difference (∆E_diabatic_) between donor and acceptor states would be relevant to ∆*E*_½_. Table 4 lists the calculated energy difference ∆E_diabatic_ for compounds 1 and 2. It can be seen that for compound 1 ∆E_diabatic_ is only 1.3 kcal/mol, suggesting that the neutral 2,5-diethynylpyridine bridge does not provide significant perturbation in the redox potentials of the two ferrocenes, because of the relatively weak electronic coupling. In contrast, for compound 2 the energy difference between the two diabatic states is rather remarkable (∆E_diabatic_ = 5.7 kcal/mol) indicating a differentiation of the redox potential between the two ferrocenyl groups.

**Table 4:** Calculated energy difference (∆E_diabatic_) between donor and acceptor states for compounds 1a and 2a.

Compound	∆E_diabatic_ (kcal/mol)
1	1.3
2	5.7

Another ferrocene-based bridging unit that may possess current-switching capabilities is the oxazole bridge [47]. It is interesting that in this system the oxazole bridge behaves quite differently from the 2,5-diethynylpyridine examined in this work: at the neutral state of this system the two ferrocenes are in good electronic communication. However, after protonation of the oxazole bridge only one redox peak was observed in voltammetric measurements. It has been proposed that this behavior may be ascribed to a reduction in the basicity of the oxazole nitrogen upon oxidation of the first ferrocene, which then leads to deprotonation of the bridge prior to the oxidation of the second ferrocene [47]. Our CDFT calculations for this system (See Table 5) show that both neutral and protonated species have moderate couplings, making them along the borderline between class I and class II compounds in the Robin-Day classification. The integration of this system into nanoscale electronic devices requires further investigation and is the subject of future work.

**Table 5:** Calculated electronic coupling for Fc-oxazole-Fc systems.

Fc-oxazole-Fc state	*H*_ab_ (kcal/mol)	
	Gas Phase	CH_2_Cl_2_ solution
Neutral	1.81	1.04
Protonated	1.57	0.80

## 4. Conclusions

In this work, the DFT, TDDFT, and CDFT methods were employed to study the geometries, electronic structures, optical transitions, and electronic couplings of an experimentally proposed ferrocene-based molecular diode bridged by the conjugated 2,5-diethynylpyridyl unit. The optimized geometries from the DFT calculation, as well as the absorption spectra from the TDDFT calculations are in good agreement with experimental data. The nature of the electronic transitions responsible for the UV-vis spectroscopy was discussed based on the corresponding molecular orbitals. For compound 2, the low-energy absorption maximum is dominated by the HOMO-LUMO transition that is characterized by a MLCT process. For compound 1, it is mainly a d-d transition within the same ferrocenyl group, plus a small contribution of the MLCT characteristic. The difference in the nature of the optical transition suggests that compounds 1 and 2 may exhibit different properties as effective nanoelectronic building blocks.

A more quantitative description of the electronic communication between the two ferrocenyl groups in compounds 1 and 2 was provided by the CDFT calculations, in which donor and acceptor states of a bridge-mediated electron transfer reaction were explicitly defined and their coupling matrix element was computed accordingly. The computational results indicated that the electronic communication between the two ferrocenyl groups through the 2,5-diethynylpyridyl bridge was along the borderline of the class I and II mixed-valence compounds. As a result, conduction through such a molecular device likely proceeded via an electron hopping mechanism. For a neutral 2,5-diethynylpyridyl bridge the electron transport might occur along both forward and reverse potential bias; whereas when a positive charge is present on the nitrogen atom of the pyridyl ring, the energies/redox potentials in the two ferrocenyl groups might be differentiated, leading to the formation of an energetic barrier for electron transfer when a forward bias was applied. The theoretical findings are also consistent with experimental results [32].

In summary, the theoretical studies reported herein provide mechanistic interpretations for varied experimental measurements, which help advance our understanding of the underlying electron transfer processes. It is anticipated that similar strategies may be applied in the design of new electronic materials, which is the subject of our future work.
